# Reduced sexual size dimorphism in a pipefish population where males do not prefer larger females

**DOI:** 10.1002/ece3.5760

**Published:** 2019-11-01

**Authors:** Mário Cunha, Nídia Macedo, Jonathan Wilson, Gunilla Rosenqvist, Anders Berglund, Nuno Monteiro

**Affiliations:** ^1^ CIBIO/InBIO Centro de Investigação em Biodiversidade e Recursos Genéticos Universidade do Porto Vairão Portugal; ^2^ CIIMAR Centro Interdisciplinar de Investigação Marinha e Ambiental Universidade do Porto Porto Portugal; ^3^ Wilfrid Laurier University Waterloo Ontario Canada; ^4^ Department of Biology CBD, NTNU Trondheim Norway; ^5^ Department of Earth Sciences Blue Centre Gotland Uppsala University Uppsala Sweden; ^6^ Department of Ecology and Genetics/Animal Ecology Uppsala University Uppsala Sweden; ^7^ Departamento de Biologia Faculdade de Ciências da Universidade do Porto Porto Portugal; ^8^ Faculdade de Ciências da Saúde CEBIMED Universidade Fernando Pessoa Porto Portugal

**Keywords:** embryonic development, male pregnancy, postcopulatory selection, sexual selection, Syngnathidae

## Abstract

Within a species' distribution, populations are often exposed to diverse environments and may thus experience different sources of both natural and sexual selection. These differences are likely to impact the balance between costs and benefits to individuals seeking reproduction, thus entailing evolutionary repercussions. Here, we look into an unusual population (Baltic Sea) of the broadnosed pipefish, *Syngnathus typhle*, where males do not seem to select females based on size and hypothesize that this pattern may derive from a reduction in direct benefits to the male. We further hypothesize that if larger females do not persistently secure a higher reproductive success, either through pre‐ or postcopulatory sexual selection, a decrease in sexual size dimorphism in the Baltic population should be apparent, especially when contrasted with a well‐studied population, inhabiting similar latitudes (Swedish west coast), where males prefer larger females. We found that, in the Baltic population, variation in female quality is low. We were unable to find differences in abortion rates or protein concentration in oocytes produced by females of contrasting sizes. Direct benefits from mating with large partners seem, thus, reduced in the Baltic population. We also found no evidence of any postcopulatory mechanism that could favor larger mothers as embryo development was unrelated to female size. While female size can still be selected through intrasexual competition or fecundity selection, the pressure for large female body size seems to be lower in the Baltic. Accordingly, we found a noticeable decrease in sexual size dimorphism in the Baltic population. We conclude that, although far from negating the significance of other selective processes, sexual selection seems to have a decisive role in supporting pipefish sexual size asymmetries.

## Graphical abstract

We look into the mechanisms supporting the evolution and maintenance of sexual size dimorphism in pipefish. We found that, in a population where males do not choose mating partners based on size, variation in female quality is low which renders male preference for larger partners pointless. Also, we were unable to find any postcopulatory mechanisms acting on the male's marsupium (e.g., half‐sib rivalry), that would favor larger females. Our results showcase the significant impact of sexual selection, at least in pipefish, in supporting sexual size dimorphism.

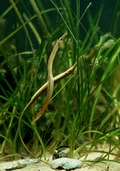

## 1 INTRODUCTION

Due to local or geographically widespread variation in environmental conditions, one can assume that the price of securing the best possible mating partner, and the extension of the ensuing rewards, likely differs throughout the distribution of a species. For instance, in gray seals, the increased female mobility in drier seasons was shown to reduce the dominant male's ability to monopolize matings (Twiss, Thomas, Poland, Graves, & Pomeroy, 2007), altering the expected degree of polygamy and impacting effective population size. On a much wider geographical scale, interhemispheric differences in polygyny rates were observed in wrens as a consequence of alterations in migration behavior and life‐history strategies that affected male's control over female reproduction (Llambías, Jefferies, Garrido, & Fernández, 2019). As a consequence of differential selection pressures across distinct environments, sexual selection intensity is expected to fluctuate (Boughman, 2001; Mobley & Jones, 2009; Monteiro, Carneiro, et al., 2017; Monteiro, Cunha, et al., 2017).

To further expand our understanding of selection responses to distinct environmental conditions, we must also take into account processes occurring after mating, as these can act in conjunction or in opposition to premating processes such as mate choice or competition for mating opportunities (Evans & Garcia‐Gonzalez, 2016). Although our understanding of pre‐ and postcopulatory selection expanded in recent years (Eberhard, 2009), information on taxa with nonconventional sex roles (Cunha, Berglund, Mendes, & Monteiro, 2018; Paczolt & Jones, 2010; Rose, Paczolt, & Jones, 2013) will certainly force us to keep looking beyond the better fleshed out hypotheses.

The fish family *Syngnathidae* (seahorses, seadragons, pipefishes, and pipehorses) has a truly unique form of paternal care: male pregnancy. Females transfer oocytes into the male's brood pouch or incubating surface, where they are fertilized and offspring development is supplemented by paternal contributions (Kvarnemo, Mobley, Partridge, Jones, & Ahnesjö, 2011; Monteiro, Almada, & Vieira, 2005; Stolting & Wilson, 2007). The brood pouch functions as a “placenta‐like” structure where nutrients are transferred between the father and developing embryos (Kvarnemo et al., 2011; Ripley & Foran, 2008; Sagebakken, Ahnesjö, Mobley, Braga Goncalves, & Kvarnemo, 2009). Additionally, in many syngnathid species, the choosier males mate with several females and prefer to do so with the larger and more ornamented (Mobley, Small, & Jones, 2011; Rosenqvist & Berglund, 2011; Silva, Vieira, Almada, & Monteiro, 2007). Thus, the combination of male pregnancy (Stolting & Wilson, 2007), sex‐role reversal (Berglund, Widemo, & Rosenqvist, 2005) and multiple matings observed in many species (Mobley, Kvarnemo, et al., 2011), provides a unique opportunity to further explore the topic of pre‐ and postcopulatory sexual selection. Indeed, postcopulatory selection has already been described in pipefish (*Syngnathus scovelli* and *S. abaster*), with males reducing investment in embryos from less preferred females to potentially save resources for future reproductive events (Cunha et al., 2018; Paczolt & Jones, 2010). Other postcopulatory selection mechanisms, such as female–female competition within the male's brood pouch in the form of sibling rivalry, as hinted by Ingrid Ahnesjö (1996), remain to be fully uncovered.

**Figure 1:**
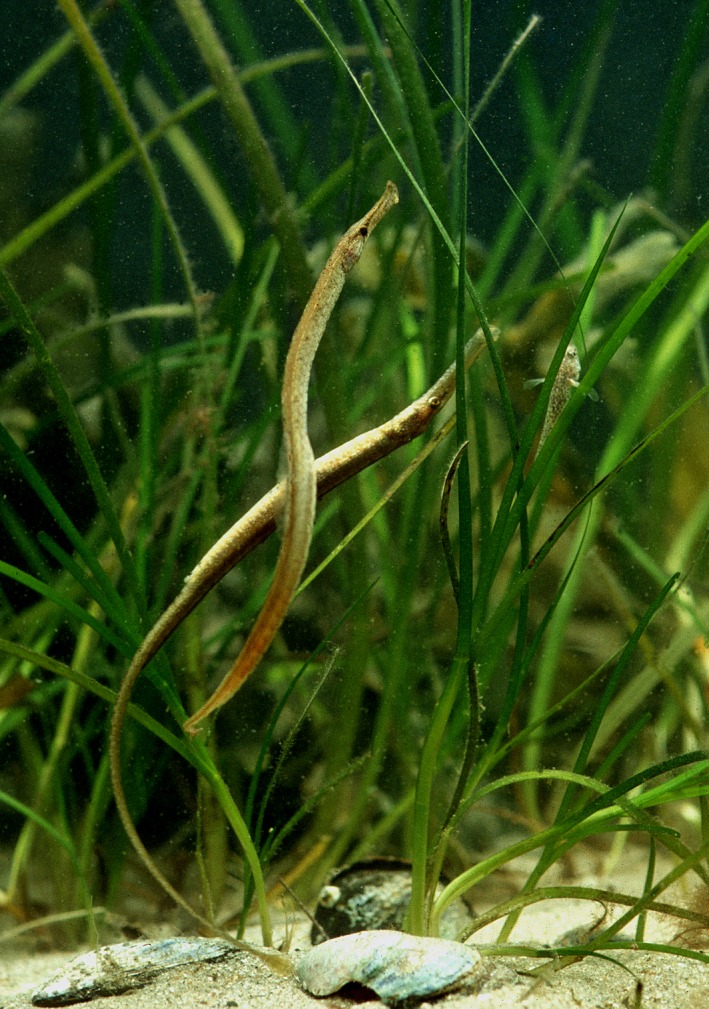
Mating broadnosed pipefish (male at the front)

The broadnosed pipefish, *Syngnathus typhle* (Figure 1), has a distribution ranging from the Mediterranean up to the Baltic Sea, thus experiencing a wide range of temperature regimes (Rispoli & Wilson, 2007) which are known to differentially influence male and female reproductive potential (Ahnesjö, 1995). As in most fish, larger *S. typhle* females tend to be more fertile, producing larger and protein richer eggs (Braga Goncalves, Ahnesjö, & Kvarnemo, 2011; Braga Goncalves et al., 2010). This observation supports the described male preference for larger females, persistently observed in one of the most studied *S. typhle* populations, located on the west coast of Sweden, in the North Sea (Berglund, 1994; Berglund, Rosenqvist, & Svensson, 1986a; Berglund et al., 2005). Remarkably, in a population located at roughly the same latitude, but in the Baltic Sea (east coast of Sweden), males were found not to discriminate females based on size (Sundin, Rosenqvist, & Berglund, 2013). Since female size is a trait usually selected by males in *S. typhle*, as well as in other pipefish species (Flanagan, Johnson, Rose, & Jones, 2014; Monteiro, Carneiro, et al., 2017; Monteiro, Cunha, et al., 2017; Rosenqvist & Berglund, 2011; Silva et al., 2007), it is curious why in this particular population (see Berglund, Sundin, & Rosenqvist, 2017 for additional details on ecology and population structure), where sexual selection is expected to act strongly on females due to their higher potential reproductive rate, males do not choose mating partners based on their size.

We hypothesize that, in contrast to what is observed on the west coast of Sweden, the absence of male preference for larger females in the Baltic should result from a reduction in direct benefits to the male. As the survival prospects of offspring can be influenced by maternal effects (Marshall, Allen, & Crean, 2008), namely the amount of resources allocated into oocytes, we addressed the hypothetical reduction in male benefits by quantifying relevant properties in eggs produced by females of different sizes. Besides measuring size and mass, we also looked for asymmetries in the protein content of eggs, as proteins are integral for tissue growth (Heming & Buddington, 1988) and were shown, in pipefish, to be essential for embryo development (Haresign & Shumway, 1981). Nevertheless, although males in the Baltic do not seem to explicitly prefer larger mating partners (Sundin et al., 2013), female size can still be under positive postcopulatory selection. Given *S. typhle* polygynandrous mating system (Rispoli & Wilson, 2007), where males often receive eggs from different females, postcopulatory sexual selection operating within the male's marsupium (e.g., male cryptic choice or half‐sibling rivalry) could still favor embryos from larger mothers, in half‐sib broods. If embryo development is affected by simpler processes, such as mating order (see Braga Gonçalves et al., 2015), female size can still be selected if larger females are recurrently able to secure the most rewarding time slot when mating (e.g., mating first). This later scenario seems less likely as the short time elapsed between sequential matings has been considered insufficient, at least in the Swedish west coast population (Mobley, Kvarnemo, et al., 2011), to support developmental asymmetries in the brood. Ultimately, if larger females do not persistently secure a higher reproductive success, either through pre‐ or postcopulatory sexual selection, we can predict a decrease in sexual size dimorphism in the Baltic population when contrasted with that from the west coast. To address these questions, we analyzed embryo growth patterns in full‐ and half‐sib broods, looking for indicators that might suggest male cryptic choice or half‐sibling rivalry in pregnancies harboring offspring from different size females. We also evaluated the contemporary degree of sexual size dimorphism in the broadnosed pipefish from both the Baltic and Sweden's west coast.

## 2 MATERIAL AND METHODS

### 2.1 Sampling and handling

The experiment was conducted at Ar Field Station on Gotland, Sweden, between May and July 2015 (permit Dnr. S15–13, Swedish Board of Agriculture). Broadnosed pipefish were caught with a beam trawl in eelgrass meadows in the central Baltic Sea on the coast of Gotland (57**°**44′20″ N, 18**°**57′40″ E). Fish were transported to the field station in isothermal containers with aeration. Once in the field station, fish were transferred to holding tanks (650 L) and separated by sex. The tanks had flow‐through water from the Baltic Sea. Temperature, salinity, and photoperiod followed natural conditions, with natural light being supplemented with artificial light bulbs. Fish were fed three times a day with live *Artemia *sp. at different development stages, together with live and frozen *Mysis *sp. All the tanks were environmentally enriched with artificial eelgrass to ensure easy access to refuges.

### 2.2 Female size and oocyte quality

We performed simple linear regressions to establish how female size relates to female weight and several oocyte variables (number, weight, size, and protein content), using data from 17 females. These females were caught before the onset of the breeding season and their gonads continued to mature in the laboratory, thus ensuring that the measured oocytes belonged to the first mature batch of that year (see Begovac & Wallace, 1988). We started by euthanizing females with an overdose of MS222 and excising the gonads. The eviscerated carcass was dried for 72 hr at 70°C to obtain dry mass. The gonads and oocytes were photographed under a stereomicroscope which allowed for oocyte counting and morphometric analysis (we measured 10 oocytes per female, with the exception of one female where only eight oocytes were mature). The oocytes were frozen at −20°C. Frozen eggs were then separated and individually homogenized in ice‐cold 50 µl of SEID buffer (0.1% sodium deoxycholate, 150 mM sucrose, 10 mM EDTA, 50 mM imidazole, pH 7.3) using a Precellys24 bead homogenizer (Bertin) at 6,800 rpm for 2 × 10 s. The homogenate was centrifuged at 17 000 × g for 5 min at 4°C and the supernatant processed according to the instructions of the Experion^TM^ Pro260 Analysis Kit (Bio‐Rad) using the included reagents. Processed samples were loaded onto protein chips and analyzed using a Bio‐Rad Experion^TM^ automated electrophoresis system and accompanying software (v3.20).

### 2.3 Mating experiment

The mating experiment started from the moment we found the first pregnant male in the wild (visits to the sampling site were regularly conducted to detect the onset of the breeding season). Mating took place in smaller barrels (200 L) with the same conditions described for the holding tanks. Mating pairs were assigned to an individual barrel and, at the end of the mating experiment, pregnant males of the day were assigned to a larger holding tank. Experimental trials consisted of four treatments: (a) males mated with a single small female (SM_S_; Single Mated with Small mating partner) or (b) males mated with a single large female (SM_L_). These first two treatments produced full‐sib broods; (c) males mated with a small female followed by a large female (MM_S‐L_, Multi‐Mated with a Small female followed by a Large female) and (d) males mated with a large female followed by a small female (MM_L‐S_). These last two treatments produced half‐sib broods.

Size classes (large or small) were established based on female standard body length data from this population (14.14 ± 1.43 cm, mean ± standard deviation, *N* = 277; data kindly contributed by J. Sundin). We used half of the standard deviation as the buffer interval to separate size classes. Small females were less than 14.7 cm (13.41 ± 0.85 cm; range: 11.4–14.7 cm, *N* = 39) and large females were greater than 15.4 cm in length (16.55 ± 0.72 cm; range: 15.4–17.9 cm, *N* = 36). Males (14.79 ± 0.88 cm; range: 13–17.1 cm, *N* = 53) and females were measured before the experiment. To assess size or weight differences between the six female groups (SM_S_, SM_L_, large ♀ in MM_L‐S_, small ♀ in MM_L‐S_, large ♀ in MM_S‐L_, small ♀ in MM_S‐L_) we conducted one‐way ANOVAs, followed by Newman–Keuls post hoc tests (NK). Similarly, we looked for male size differences between the four male groups (SM_S_, SM_L_, MM_L‐S_, MM_S‐L_) with one‐way ANOVAs, followed by Newman‐Keuls post hoc tests (NK). Data assumptions were met, as verified with Shapiro–Wilk (normality) and Levene (homogeneity of variances) tests.

Males with access to a single female were allowed to mate and after the first eggs were transferred (each tank was checked hourly for mating events for a period of 16 hr every day) the female had 24 additional hours to transfer more eggs after which she was removed. In the multi‐mated treatments, the first female was allowed to fill the male's pouch to approximately half (easily determined through visual estimation by an experienced observer). The female was then removed and a second, different‐sized female was added and allowed to fill up the other half of the male's pouch within the following 24 hr. After males got pregnant (either from one or two females) they were transferred to a holding tank together with the other pregnant males from that day (650 L tanks). Immediately after the females mated, we removed them from the tank, euthanized, dissected and the carcass dry mass was determined as described above.

As is many other teleosts (Blaxter, 1988), pipefish embryo development rate is intimately dependent on temperature (e.g., Monteiro, Almada, & Vieira, 2003; Silva, Monteiro, Almada, & Vieira, 2006). Since our tanks followed natural temperature conditions (flow‐through system with water from the Baltic), we established the extent of the pregnancy interval in degree‐days (sum of daily average water temperature over a time interval, in days) to scale development to the physiology that drives ectotherm growth (see Neuheimer & Taggart, 2007). We kept pregnant males in the holding tanks for 300 degree‐days (°C · day). During this period, the embryos are still inside the chorion so egg position in the pouch reflects, in multi‐mated males, the order by which females deposited their oocyte batches (first female on the bottom and second at the top of the brood pouch, as later confirmed by parentage analysis). At the selected time point, it is already possible to measure embryo length and the remaining yolk, and perform parentage analysis.

We euthanized males and counted the number of embryos within the male's pouch while recording their relative position. Under a stereomicroscope, we carefully dissected the eggs and removed the embryos. We calculated embryo relative survival for each brood by dividing the number of developed embryos by the total (normal developing embryos plus abortions). Approximately half of the embryos from each brood were photographed with a digital microscope (Dino‐lite AM7013NT) alongside a ruler for measurements of embryo length and yolk area and then preserved in 96% ethanol. Yolk area was calculated using the formula of an ellipse, as its photographed 2D shape was rarely circular.

For parentage analysis, DNA was extracted from females, pregnant males and developing embryos (≈38% of the photographed embryos, randomly selected) using the Genomed JETquick tissue DNA spin kit. Samples were amplified for two highly polymorphic microsatellite markers (typh04 and typh18, Jones, Rosenqvist, Berglund, & Avise, 1999) considered sufficient given the small number of possible mothers. For instance, even in the tank where we housed more pregnant males, the likelihood of making a correct assignment was extremely high (Pe = 0.99). For both microsatellites, reaction tubes (10 μl) contained 5 μl MasterMix (Alphagene), 1 μl H2O, 0.8 μM primer, 0.8 mM fluorescently labeled tail. PCR setup started with an initial denaturation of 94°C for 15 min, followed by 30 cycles with 94°C for 30 s, 58°C annealing for 30 s with 72°C extension for 30 s. Then we ran 10 cycles with 94°C for 30 s denaturation, 53°C annealing for 30 s, and 72°C for 30 s for the fluorescent tail. Final extension was 72°C for 15 min. All PCRs were conducted in a BIO‐RAD MyCyclerTM Thermal Cycler (Applied Biosystems). Allele sizes were determined on an ABI 3100 capillary sequencer (Applied Biosystems) using LIZ 75–450 as size standard (Applied Biosystems). The size of each fragment was then determined in Peak Scanner Software v1.0 (Applied Biosystems). Due to the low number of mothers in half‐sib broods, the maternity assignment was done visually, using the exclusion method (Jones, Small, Paczolt, & Ratterman, 2010).

We looked for differences in the number of embryos (data was squared for normality) between the four treatments (SM_S_, SM_L_, MM_L‐S_, MM_S‐L_), using a one‐way ANOVA, followed by an NK test. A similar approach was used with embryo length and yolk size. When searching for asymmetries in abortion rates between our four treatments, as data could not be normalized, we performed a Kruskal–Wallis ANOVA, followed by a Wilcoxon‐matched pairs test. We also performed additional linear regression analysis on (a) brood size and number of aborted embryos, as well as (b) yolk area and embryo size, within each of the treatments.

To avoid data dependence on multi‐mated treatments, we averaged embryo length of the first female transferring the eggs (either small or large) and subtracted the average embryo length of the second female. If embryos from the first female are larger than those of the second female, the result would be positive and significantly different from zero (one‐sample *t* test), irrespectively of female size, highlighting the importance of mating order. If, instead, female size is the most important factor, the obtained value will be positive when large females mate first and negative when small females are the first to mate. In this instance, when comparing the two multi‐mated treatments (with either large or small females mating first), we will be contrasting predominantly positive values in both groups if mating order is a key factor in development speed, or two sets of values with different signs if female size is critical. In this last case, a mating order effect can be detected if the absolute (i.e., modulus) average values are similar and different from zero.

### 2.4 Sexual size dimorphism

To analyze the contemporary degree of sexual size dimorphism in pipefish from the east (Baltic: data from J. Sundin) and west coast of Sweden (data from Berglund & Rosenqvist, 1990), we gathered length measurements from a total of 974 pipefish (East coast: 277F and 208M; West coast: 263F and 226M). We then conducted a Kruskal–Wallis ANOVA (data was not normal and could not be transformed) to compare female and male sizes from the East and West coast (4 groups), followed by a Wilcoxon rank‐sum test (with Holm adjustment). Finally, we calculated the sexual size dimorphism (female – male standard length) for both locations and also contrasted male size (as a proportion of female size) between the Baltic and Sweden's west coast, using a *Z*‐score test.

## 3 RESULTS

### 3.1 Female size and oocyte quality

Regression analysis showed that female length (14.91 ± 1.22 cm, average ± standard deviation, *N* = 17) was found to be positively correlated with body mass, in the Baltic. This pattern remains unchanged if we use dry (*R* ^2^ = .77, *p* < .001; average female dry weight: 0.17 ± 0.04 g, *N* = 17) or wet body mass (*R* ^2^ = .74, *p* < .001; average female wet weight: 1.25 ± 0.33 g, *N* = 17). Although larger females produce more (*R* ^2^ = .30, *p* < .05) and heavier oocytes (*R* ^2^ = .35, *p* < .05) than smaller females (average female oocyte number: 94.29 ± 50.91, *N* = 17; average female oocyte weight: 2.14 ± 0.50 mg, *N* = 17), we were unable to find a relationship between body length and either oocyte diameter (1.45 ± 0.08 mm, *N* = 17; *R* ^2^ = .19, *p* = .08) or oocyte protein concentration (631.88 ± 191.57 ng/egg, *N* = 17; *R* ^2^ = .04, *p* = .43). In fact, oocyte protein content did not correlate with any of our measured variables. Specifically, we found no apparent link between protein content and either oocyte size (*R* ^2^ = .003, *p* = .83, *N* = 17) or oocyte mass (*R* ^2^ = .002, *p* = .84, *N* = 17).

### 3.2 Mating experiment

Males did not differ in size in the four treatments (SM_S_, 14.84 ± 1.05 cm, *N* = 14; SM_L_, 14.56 ± 0.86 cm, *N* = 10; MM_S‐L_, 14.60 ± 0.66 cm, *N* = 10; MM_L‐S_, 15.05 ± 1.01 cm, *N* = 10; one‐way ANOVA, *F*(3,40) = 0.62, *p* = .60). However, as intended, females did vary, both in length (one‐way ANOVA, *F*(5,58) = 50.35, *p* < .001) and dry body mass (one‐way ANOVA, *F*(5,58) = 57.56, *p* < .001). Expectedly, all small female groups were similar (13.46 ± 0.87 cm; 0.13 ± 0.02 g, *N* = 34) and so were the large female groups (16.54 ± 0.68 cm; 0.25 ± 0.04 g, *N* = 30), as verified by a NK test.

**Figure 2:**
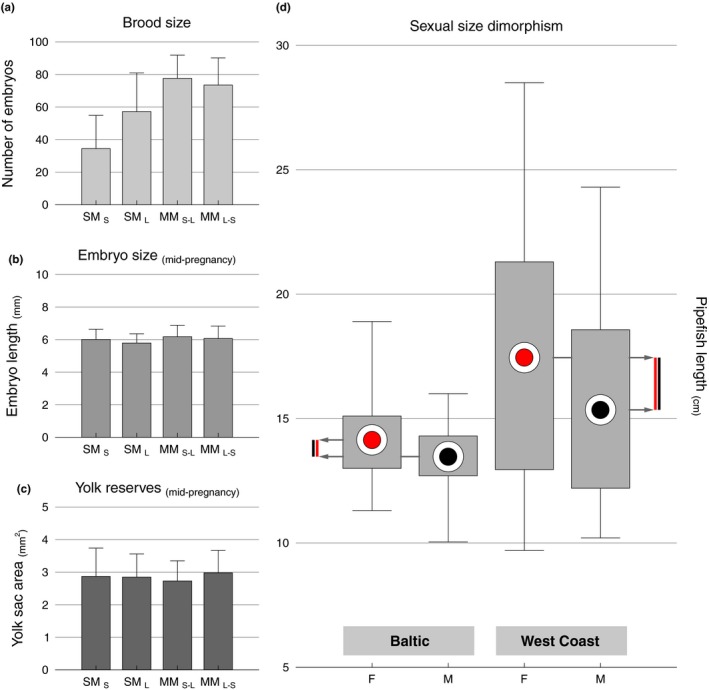
Average brood size (a), embryo size (b), and yolk area (c) in the four experimental treatments (error bars depict standard error; SM_S_—male mated only with a small female, SM_L_—male mated only with a large female, MM_S‐L_—male mated with a small and then a large female, MM_L‐S_—male mated with a large and then a small female)—data from the Baltic population. On the right (d), male and female sizes from the Baltic and West coast of Sweden (circles indicate the mean, boxes show 25th and 75th percentiles, and whiskers show maximum and minimum values. Vertical bars on both margins help to visually compare the sexual size dimorphism on the two populations)

We found significant differences in embryo number between the four treatments (one‐way ANOVA, *F*(3,40) = 11.16, *p* < .001; Figure 2a). Post hoc test (NK) revealed that males mating only with a small female (SM_S_) carried fewer embryos than all other groups (SM_S_: 34.5 ± 20.40 embryos). Multi‐mated males carried similarly high embryo numbers (MM_S‐L_: 77.60 ± 14.26; MM_L‐S_: 73.50 ± 16.69 embryos). Males mated with a single large female (SM_L_: 57.20 ± 23.76 embryos) presented intermediate‐sized broods.

Aborted embryos (%) did not differ between treatments (Kruskal–Wallis ANOVA, H(3, *N* = 44) = 2.89, *p* = .41). When looking exclusively at multi‐mated males (MM_S‐L_ or MM_L‐S_), we found no difference in the number of aborted embryos between the first and second female (Wilcoxon‐matched pairs test; MM_S‐L_: *Z* = 0.42, *p* = .67; MM_L‐S_: *Z* = 0.42, *p* = .67). Also, we were unable to find a relationship between brood size and the number of aborted embryos within any treatment (SM_S_: *R* ^2^ = .01, *p* = .74; SM_L_: *R* ^2^ = .18, *p* = .22; MM_S‐L_: *R* ^2^ = .05, *p* = .53; MM_L‐S_: *R* ^2^ = .24, *p* = .15).

Embryo length was found to be similar among all four treatments (one‐way ANOVA, *F*(3,60) = 075, *p* = .53; SM_S_: 6.01 ± 0.63 mm; SM_L_: 5.79 ± 0.57 mm; MM_S‐L_: 6.18 ± 0.70 mm; MM_L‐S_: 6.07 ± 0.76 mm; Figure 2b), even when multi‐mated males were divided into two separate groups, according to contributing mother (one‐way ANOVA, *F*(5,58) = 0.78, *p* = .57). Likewise, the dimensions (area) of the yolk sac still visible in the developing embryos was similar among treatments (one‐way ANOVA, *F*(3,59) = 0.81, *p* = .49; SM_S_: 2.87 ± 0.87 mm^2^; SM_L_: 2.85 ± 0.71 mm^2^; MM_S‐L_: 2.60 ± 0.85 mm^2^; MM_L‐S_: 2.98 ± 0.69 mm^2^; Figure 2c), even after subdividing multi‐mated males (one‐way ANOVA, *F*(5,57) = 0.71, *p* = .61). We found a positive correlation between yolk area and embryo size, but only in the two treatments were males mated with a single large or small female (SM_S_: *R* ^2^ = .30, *p* < .05; SM_L_: *R* ^2^ = .47, *p* < .05). In the two treatments where males mated with more than one female, no such relationship was observed (either when considering the complete brood or when subdividing by each of the contributing mothers).

In multi‐mated males, after subtracting average embryo lengths from the two contributing females (1st minus 2nd), we detected that the obtained values were predominantly positive (17 in 20) and significantly different from zero (one‐sample *t* test, *t* = 4.68, DF = 19, *p* < .001), thus highlighting the relevance of mating order (positive values show that embryos from the first egg batch grew more than those from the second batch). The resulting patterns stand even when we analyzed both treatments separately.

### 3.3 Sexual size dimorphism

A Kruskal–Wallis ANOVA revealed that there was a significant difference in pipefish length [*H*(3, 974) = 83.72, *p* < .001; Figure 2d] between the four groups (males and females from the west coast, males and females from the Baltic). All four groups differed from each other, with females being larger than males and pipefish from the Baltic being smaller than those from the west coast. Male size (as a proportion of female size) differed among locations (*Z*‐score test, *Z* = 3.91, DF = 1, *p* < .001; 0.88 in the west and 0.95 in the Baltic), with larger sexual size dimorphism in the west coast (2.11 cm, female–male average length difference) when compared to that registered in the Baltic (0.67 cm, see Figure 2d).

## 4 DISCUSSION

Discriminating among potential mates of varying quality can be vital for fitness enhancement. Therefore, it is common to observe the recurrent preference for larger mating partners which can directly translate into higher reproductive success (Kraushaar & Blanckenhorn, 2002; Wootton, 1979). In syngnathid species, where males typically are the choosier sex, larger females are often preferred because body size frequently correlates with fertility (Berglund, 1991; Wilson, 2009). Accordingly, in one of the most well‐studied broadnosed pipefish populations, a male preference for larger females has been persistently documented (Berglund, 1994; Berglund & Rosenqvist, 2001; Berglund et al., 1986a). These larger females are known to produce more, larger and protein richer eggs (Berglund et al., 1986a; Berglund, Rosenqvist, & Svensson, 1986b; Braga Goncalves et al., 2011), thus helping produce fitter offspring (Ahnesjö, 1992a, 1992b). Although size is undoubtedly not the only cue used by pipefish males (Berglund & Rosenqvist, 2001; Cunha, Berglund, & Monteiro, 2017; Monteiro, Carneiro, et al., 2017; Monteiro, Cunha, et al., 2017; Roth, Sundin, Berglund, Rosenqvist, & Wegner, 2014), by preferentially selecting larger mating partners males are not only increasing their fitness but also stimulating a significant relationship between sexual selection and sexual size dimorphism. Accordingly, in the *S. typhle* population where males typically prefer to mate with larger females, we found marked female‐biased sexual size dimorphism.

As hypothesized, in the broadnosed pipefish Baltic population, where males do not seem to select mating partners based on their size (Sundin, Berglund, & Rosenqvist, 2010), we found that variation in female quality is apparently low. Although larger females seemed able to produce more and heavier oocytes, these were otherwise similar to those laid by smaller females. Notably, we found no difference in protein concentration between oocytes derived from mothers of contrasting sizes. As proteins are a vital resource for embryo development (Lubzens, Bobe, Young, & Sulivan, 2017), the registered similarities support our observations of comparable abortion rates between treatments and identical embryo growth rates. Thus, unless males have scarce access to mating partners (which is not the case in this population, see Berglund et al., 2017) and favor a quick filling of the marsupium by a low number of contributing females, there seems to be little justification for a preference for large females (as opposed to what is observed on the Swedish West Coast where males accrue direct benefits from choosing females based on their size). By apparently neglecting mating partner size, males may reduce searching effort costs given that they will receive oocytes of similar quality (e.g., identical protein concentrations) from both large and small females. Direct benefits from mating with large partners (in the form of egg quality) seem, thus, absent in the Baltic population.

Despite the lack of male mate choice size preferences, female size could still be under positive natural selection (e.g., fecundity increases with size) and postcopulatory selection. Mechanisms such as male cryptic choice or half‐sibling rivalry could still favor larger mothers. We found no discernible evidence of any of these mechanisms in action in the Baltic population. For instance, if embryos from larger females have a competitive advantage over embryos from smaller females, one could expect embryo survival to be lower in multi‐mated males. However, embryo survival was found to be similar among all our experimental groups. This lack of differences in embryo survival also contrasts with observations made in the Swedish west coast population where multi‐mated males had broods with higher relative embryo survival when compared to single‐mated males (Sagebakken, Ahnesjö, Braga Goncalves, & Kvarnemo, 2011). We did, however, uncover an unexpected result that may be due to sibling rivalry within the marsupium, as suggested by Ingrid Ahnesjö (1996). Unexpectedly, as growing necessarily consumes resources, larger embryos within full‐sib broods (of large or small females alike) still retained larger yolk sacs. This positive correlation between embryo length and remaining yolk could suggest that these larger embryos already had more reserves from the start or that they are more efficient in capturing paternal contributions, thus being able to use their yolk reserves at a slower pace. These results certainly do not prove the existence of sibling rivalry (as we do not know why this pattern is absent from multi‐mated males) but deserve notice nevertheless.

Although we did not measure offspring viability after parturition and cannot make conclusive inferences on fitness (our observations were conducted mid‐pregnancy), we found that embryos from larger mothers did not show any discernible difference from those coming from smaller females. In fact, our data suggest that embryo development derives primarily from mating order. Despite the short interval elapsed between two consecutive copulations, and independently from the size of the mother, the first eggs in the marsupium gave rise to embryos that were noticeably ahead in terms of development. Consequently, while no evident postcopulatory mechanism was detected, mating order could be viewed as an additional explanation for stronger sexual selection acting upon females, in the form of female–female competition. With a higher reproductive rate (Berglund et al., 2017), females are expected to compete for early access to high‐quality mates (Berglund & Rosenqvist, 2009). In the wild, larger females probably have a competitive advantage over smaller females to mate first. This competitive advantage has already been documented in *S. typhle* (Bernet, Rosenqvist, & Berglund, 1998), and also in other syngnathid species (Silva, Vieira, Almada, & Monteiro, 2010). While female size can still be selected through intrasexual competition or fecundity selection (see Winkler, Stölting, & Wilson, 2012), the pressure for large female body size seems partially reduced in the Baltic as males have less to gain from choosing larger females. As a probable result, sexual size dimorphism is also lower in the Baltic.

Several distinct nonexclusive processes have often been referred to as indispensable for the evolution and maintenance of sexual size dimorphism (Blanckenhorn, 2005; Hedrick & Temeles, 1989). For instance, fecundity selection can favor increased female size if larger bodies translate into higher reproductive investments (Olsson, Shine, Wapstra, Uivari, & Madsen, 2002; Rispoli & Wilson, 2007; Winkler et al., 2012). Also, sexual selection can uphold larger body sizes on the sex most prone to compete for mating opportunities (Andersson, 1994). Moreover, natural selection can also support size dimorphism by increasing differences in development periods between the sexes (Badyaev, 2002) or by avoiding extensive niche overlaps, thus reducing intersexual competition (e.g., for food; Andersson & Norberg, 1981).

In the broadnosed pipefish, the clear female‐biased sexual size dimorphism recorded in the Swedish west coast population can potentially derive from all of the above‐mentioned processes. Larger females have larger ovaries and, during a single the breeding season, have the potential to reproduce with more males and deposit more eggs (Berglund et al., 1986a, 1986b). Also, larger females have a competitive advantage over smaller subordinate females, guaranteeing access to more high‐quality males (Berglund & Rosenqvist, 2003). Thus, sexual size dimorphism in this population can be seen as resulting from the combined action of, at least, fecundity and sexual selection.

When populations respond to distinct environmental and demographic factors, mating systems are expected to vary (Mobley & Jones, 2009). Indeed, the unique conditions of the Baltic sea, specifically its low salinity (thwarted growth; Berglund et al., 2017) and eutrophication‐linked turbidity (mate choice impacted by poorer visibility; Sundin et al., 2010) both have the potential to profoundly alter *S. typhle* mating patterns and sexual selection intensity. Here, we listed the combined absence of male mate choice and any recognizable postcopulatory sexual selection mechanism operating in the marsupium. The noticeable drop in sexual size dimorphism in the Baltic population, although far from negating the significance of other selective processes, emphasizes the relevance of sexual selection in supporting size asymmetries between the sexes.

## CONFLICT OF INTEREST

The authors declare no competing interest.

## AUTHOR CONTRIBUTIONS

MC, NMa, GR, and JW performed field and laboratory work. MC, NM, and AB run data analysis. All authors helped writing the final version of the manuscript.

## Data Availability

Data deposited in Dryad (https://doi.org/10.5061/dryad.0cfxpnvx7).
